# IGRNet: A Deep Learning Model for Non-Invasive, Real-Time Diagnosis of Prediabetes through Electrocardiograms

**DOI:** 10.3390/s20092556

**Published:** 2020-04-30

**Authors:** Liyang Wang, Yao Mu, Jing Zhao, Xiaoya Wang, Huilian Che

**Affiliations:** 1 Beijing Advanced Innovation Center for Food Nutrition and Human Health, College of Food Science and Nutritional Engineering, China Agricultural University, Beijing 100083, China; 2017301010314@cau.edu.cn (L.W.); 2018306100301@cau.edu.cn (Y.M.); 2018306100426@cau.edu.cn (X.W.); 2 School of Kinesiology, Nutrition and Food Science, Rongxiang Xu College of Health and Human Services, California State University, Los Angeles, 5151 State University Dr., Los Angeles, CA 90032, USA; jzhao30@calstatela.edu

**Keywords:** prediabetes, 12-lead ECG, deep learning, high-accuracy diagnosis

## Abstract

The clinical symptoms of prediabetes are mild and easy to overlook, but prediabetes may develop into diabetes if early intervention is not performed. In this study, a deep learning model—referred to as IGRNet—is developed to effectively detect and diagnose prediabetes in a non-invasive, real-time manner using a 12-lead electrocardiogram (ECG) lasting 5 s. After searching for an appropriate activation function, we compared two mainstream deep neural networks (AlexNet and GoogLeNet) and three traditional machine learning algorithms to verify the superiority of our method. The diagnostic accuracy of IGRNet is 0.781, and the area under the receiver operating characteristic curve (AUC) is 0.777 after testing on the independent test set including mixed group. Furthermore, the accuracy and AUC are 0.856 and 0.825, respectively, in the normal-weight-range test set. The experimental results indicate that IGRNet diagnoses prediabetes with high accuracy using ECGs, outperforming existing other machine learning methods; this suggests its potential for application in clinical practice as a non-invasive, prediabetes diagnosis technology.

## 1. Introduction

Diabetes is a set of metabolic disorders pertaining to the body’s regulation of protein, fat, water, and electrolytes, caused by an absolute or relative insulin deficiency and a decreased insulin sensitivity of target tissue cells. According to the Global Diabetes Map (9th Edition) released by the International Diabetes Federation (IDF) in October 2019, approximately 463 million people worldwide have diabetes (8.3% of the global population) and this number is growing rapidly [1]. In the “Definition and diagnosis of diabetes mellitus and intermediate hyperglycemia”, published by the World Health Organization (WHO) in 2006, it was pointed out that impaired fasting glucose (IFG) is produced when fasting plasma glucose is present with a concentration of 6.1–6.9 mmol/L, but 2-h after a glucose load is of <7.8 mmol/L; impaired glucose tolerance (IGT) occurs when fasting plasma glucose levels are <7.0 mmol/L but 2-h after a glucose load are 7.8–11.1 mmol/L. The above condition is impaired glucose regulation (IGR), also known as prediabetes. It is worth noting that IGR exhibits no obvious clinical symptoms. If it remains undetected and unaddressed, 70% of patients will develop diabetes after a 9–12 year incubation period [2]. Meanwhile, they are also at increasing risk of cardiovascular and cerebrovascular diseases, diabetic nephropathy, retinopathy, and neuropathy. Fortunately, the prediabetes phase is reversible. Studies have shown that lifestyle and drug interventions in patients with IGT can reduce the risk of type 2 diabetes by 40–60% [3]. Therefore, it is particularly important to detect IGR early and take timely measures to reduce the incidence of diabetes.

Traditional blood glucose measurement is a minimally invasive test, though it is limited by many conditions, such as time, space, and materials. Climatic factors, drug use, excessive consumption of high-fat foods, long-term constipation, and insufficient drinking water can cause fasting plasma glucose levels to rise, fall, or fluctuate. Repeated measurements also increase the risk of blood loss and infection. Axelsen et al. found that pre-diabetic rats—in that diabetes was induced by a high-fat fructose diet—exhibited prolonged ventricular depolarization time, decreased conduction velocity, and increased arrhythmia during reperfusion; these were reflected in their ECGs [4]. Yang et al. analyzed a cross-section of 9988 middle-aged and elderly subjects and found that an increase in resting heart rate (RHR) was related to IGR [5]. A multi-ethnic cohort study in the United States found that when an unrecognized myocardial infarction was identified by either a pathological Q wave or a mild Q wave with ST-T abnormalities in a 12-lead ECG, the risk of myocardial infarction was higher than for people with normal fasting glucose [6]. Gudul et al. conducted routine and tissue Doppler echocardiography on IFG-afflicted, IGT-afflicted, and healthy subjects, they found that in patients with prediabetes, the atrial conduction time and P wave dispersion time measured from the ECG were longer; furthermore, the mechanical function of the left atrium was impaired after the diastolic function worsened [7].

A study using ECGs to evaluate the effects of autonomic nervous system abnormalities on prediabetes and blood glucose parameter changes on cardiovascular parameters found that the time-domain parameters of heart-rate variability were significantly reduced in patients with prediabetes. The short-term heart-rate variability parameters and blood glucose indexes also showed a significant negative correlation, indicating that parasympathetic activity of the cardiac autonomic nerve function was reduced, and that cardiac autonomic nerve dysfunction (CAND) might occur in prediabetics [8]. Moreover, some researchers have employed ECGs to conduct prospective studies of IFG, diabetes, and the development of IFG into diabetes; they found that long-term IFG can lead to accelerated RHR, ST-T changes, and arrhythmias. Among them, arrhythmia was predominately a premature atrial contraction, followed by ventricular premature contraction; thus, it is believed that heart rate measurements can be used to identify individuals with a higher risk of diabetes in the future [9]. All the above reports suggest that blood glucose levels are significantly increased in the IGR stage and that complications such as cardiovascular and diabetic microvascular diseases are already present; however, this is not typically obvious. Therefore, the recognition of small changes in the ECG can result in a preliminary diagnosis and screening of prediabetics in asymptomatic but high-risk populations, such as those who smoke or lead unhealthy lifestyles, or those with a family history of diabetes. We can thereby facilitate timely lifestyle and drug interventions and reduce these individuals’ risk of developing diabetes. 

With the evolution of artificial intelligence (AI), machine learning is becoming more widely used in precision medicine [10,11,12,13]. In a study of ECGs, [14] used deep learning algorithms to classify the heart rates of patients, achieving encouraging results. A cohort study was conducted in [15], and the ejection fraction was predicted using 12-lead ECGs and a combination of deep learning algorithms; the specificity, sensitivity, and accuracy of their system were 86.8%, 82.5%, and 86.5%, respectively, and this method could effectively detect left ventricular systolic dysfunction. Meanwhile, machine learning can also be used to extract physiological signals of the human body from ECGs. Sun et al. trained a convolutional neural network (CNN) to accurately classify sleep patterns from ECGs and respiratory signals [16]. Simjanoska et al. predicted blood pressure using ECG signals and machine learning algorithms [17]. It is worth noting that there are currently a few studies on the use of machine learning to detect diabetes through ECGs or heart rate signals [18,19,20,21], which provides a novel idea for the future promotion of non-invasive diagnostic techniques. However, as of now, we have not found any report of IGR diagnosis with this method. In the future, with the growing popularity of wearable ECG-monitoring devices [22,23], disease diagnosis and physiological examinations using machine learning will become quicker and more convenient; this will be conducive to people’s timely access to health information and allow for the early detection and treatment of diseases. 

This paper proposes IGRNet, a deep learning model that can automatically diagnose prediabetes using a 12-lead ECG. The model is a CNN with four convolutional layers and two pooling layers. To enhance the efficacy of the neural network model, we introduced and compared four activation functions (rectified linear unit (ReLU), LeakyReLU, exponential linear unit (ELU), and ClippedReLU). In our experiment, we compared IGRNet against two mainstream models commonly used in ECG research (AlexNet and GoogLeNet) and three classical machine learning algorithms (support vector machine (SVM), random forest (RF), and k-nearest neighbors (k-NN)), to highlight the excellent performance of IGRNet in this task. Next, we further classified the datasets according to the age, gender, and weight of the subjects, then performed 5-fold cross-validation using sub-datasets of the same category, to reduce the interference of irrelevant variables on the IGR diagnosis and thus improve the model. Finally, independent test sets were employed to test the capability of IGRNet, comparing other models. To our knowledge, this is the first study in which deep learning has been used to diagnose prediabetes from ECGs. 

## 2. Materials and Methods

### 2.1. Acquisition and Partitioning of Datasets 

The 12-lead ECGs (of 5 s duration) and the corresponding disease information belonging to electronic health records involved in this project were collected from three designated hospitals in Beijing, China, between 2017 and 2019. After rigorous verification of the accuracy and excluding data with incomplete variables, we obtained a total of 2251 complete case data as training sets and 663 complete case data as independent test sets, which were mainly from middle-aged and aged groups with high-risk (family history of diabetes, smoking habits, and poor eating habits, etc.). We confirmed that these samples had no clear diagnosis of cardiovascular diseases such as coronary heart disease, heart failure, severe valvular disease, etc. According to the definition of IFG and IGT in the “Definition and diagnosis of diabetes mellitus and intermediate hyperglycemia” published by the WHO (IFG: fasting plasma glucose is present with a concentration of 6.1–6.9 mmol/L but 2 h after a glucose load of < 7.8 mmol/L; IGT: fasting plasma glucose levels are < 7.0 mmol/L but 2 h after a glucose load are 7.8–11.1 mmol/L) and combining the diagnosis results of the hospital reports (We confirmed that each sample received an ECG test within a short period of time after taking the blood glucose measurement during medical examination.), we categorized the cases into those with normal blood glucose and those diagnosed with IGR (i.e., prediabetes). Previous studies have shown that factors such as weight, gender, and age may affect ECGs [24,25,26]. Therefore, according to the individual conditions of patients, the data were further categorized according to body mass index (BMI ≥ 25.0 or < 25.0), sex (male or female), and age (under 60 years old or over 60 years old); based on the above partitioning method, we divided the overall training data into seven datasets, which were named dataset_1, dataset_2, dataset_3, dataset_4, dataset_5, dataset_6, and dataset_7, respectively (where dataset_1 is the total dataset, as shown in Table 1) and overall test data into eight independent test sets, which were named test set_0, test set_1, test set_2, test set_3, test set_4, test set_5, test set_6, and test set_7, respectively (where test set_0 is the total test set and test set_1 is the mixed test set selected randomly from the total test data, as shown in Table 2). It must be emphasized that no human subjects were involved in this study. We only used licensed historical registration data in our analysis, and no members of the team were able to obtain the private information of patients when analyzing the data. This study has been approved by the Human Research Ethics Committee in China Agricultural University (approval number: CAUHR-2020003).

### 2.2. Electrocardiogram Preprocessing

**Figure 1:**
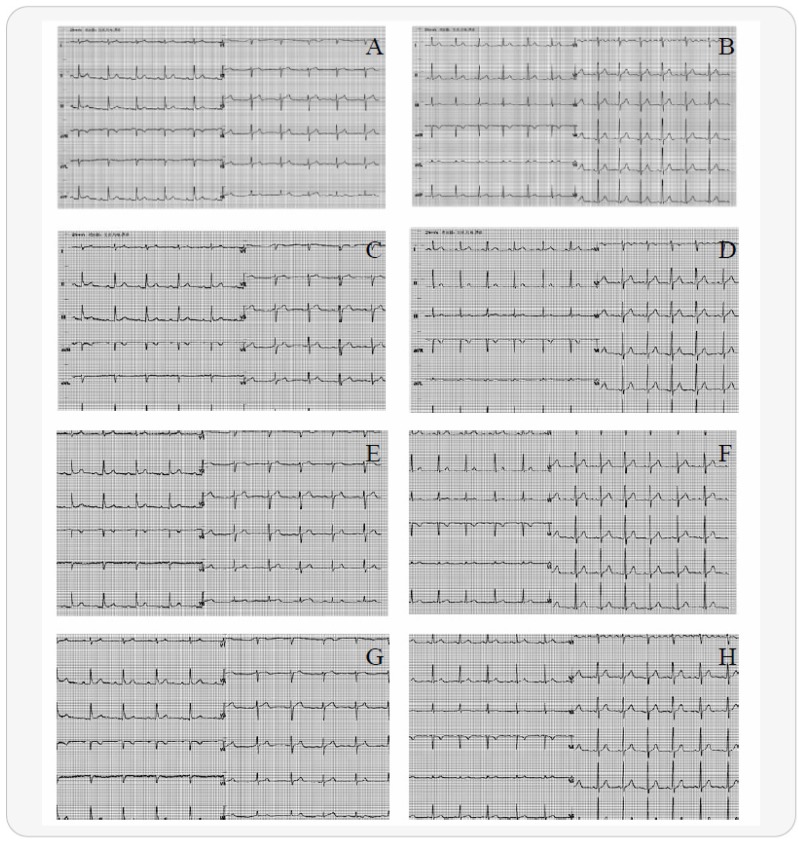
Steps of electrocardiogram (ECG) preprocessing: (**A**) ECG of a normal person; (**B**) ECG of a patient with prediabetes; (**C**,**E**,**G**) are the images after performing the left top, right bottom, and center cropping operations using (**A**), respectively; (**D**,**F**,**H**) are the images after performing the left top, right bottom, and center cropping operations using (**B**), respectively.

Preprocessing is an indispensable step in the field of computer vision. It converts initial data into a form that is suitable for computation. Existing ECG preprocessing techniques mainly involve wavelet transforms—to reduce noise, eliminate baseline drift, and data segmentation [27], which is a complex procedure. Studies have been published in which the original ECGs did not undergo a significant amount of preprocessing; instead, after performing random cropping operations, they were directly inputted into two-dimensional CNNs (2D-CNNs) in the form of grayscale images for training; this resulted in an average accuracy and sensitivity of 0.991 and 0.979, respectively [28]. Our experiment did not use traditional processing methods but instead performed data augmentation on the ECGs according to [28] (the raw data were 2-dimensional images, as shown in Figure 1A,B), eventually achieving excellent results.

There was a large imbalance between the numbers of positive and negative samples in this experiment, which could have led to poor results; thus, we introduced data augmentation technology in training sets to resolve the problem. Data augmentation is a popular method for dealing with insufficient sample sizes and sample imbalances in data mining [27,29]. For dataset_1, the unified image size was initially 500 × 300; then, the ECG images were augmented using different cropping methods (left top (Figure 1C,D), right bottom (Figure 1E,F), and center (Figure 1G,H), etc.), resulting in a fixed image size, that is 375 × 225. After that, cropped images were resized to 500 × 300. Otherwise, considering the influence of data volume on the model, we augmented dataset_2, dataset_3, dataset_4, dataset_5, and dataset_6 again after data balancing, so as to approximate the data size of dataset_1; this was convenient for subsequent comparisons (the expanded sample size is shown in Table 3). It is worth noting that the independent test sets did not be performed data augmentation operation. 

### 2.3. Model Architectures

**Figure 2:**
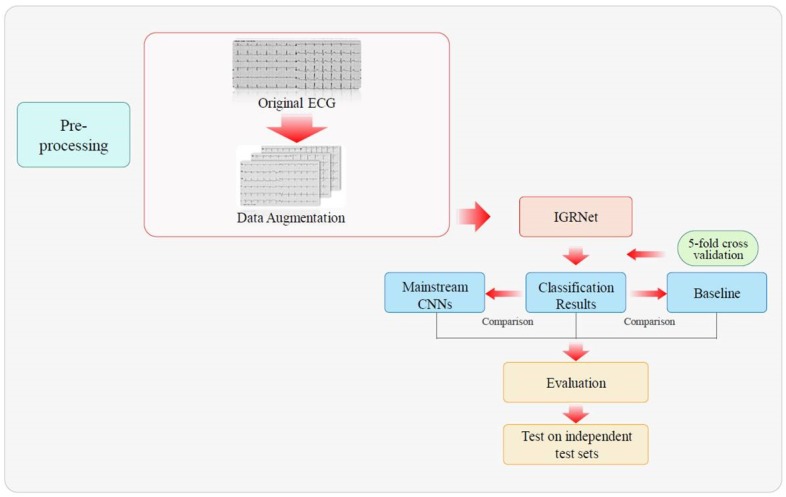
The workflow of prediabetes diagnosis.

The initial form of the input for this study was that of 12-lead ECG images with a duration of 5 s. Therefore, we considered a 2D-CNN in the deep learning models, and in the traditional machine learning models we considered using a histogram of oriented gradient (HOG) to extract ECG image features and identify them using baseline classification algorithms. CNNs are widely used in ECG intelligent diagnoses because of their layer-by-layer feature extraction and their end-to-end recognition. In this study, a new CNN architecture—referred to as IGRNet—was designed. This was compared against mainstream deep CNN models (AlexNet and GoogLeNet). Three baseline algorithms (SVM, RF, and K-NN) were also employed in this research. The entire experimental process is as shown in Figure 2.

#### 2.3.1. IGRNet

The characteristics of 12-lead ECG images are not as rich as medical images and relatively uniform in texture and color; after various attempts, the overall IGRNet architecture presented in this paper included four convolutional layers, two pooling layers, one fully connected layer, and one output layer. We set the input layer size to 128 × 128 × 3 to improve the model efficiency.

The convolution kernel is a learnable parameter in the convolution operation. In our experiments, the size of the convolution kernel was set to 5 × 5, whilst the stride of the convolution parameter was set to 1 and the padding to 2. After several verification experiments, the final number of feature maps in each convolutional layer was set as 6, 16, 120, and 250, respectively. The pooling layer is predominately used for feature dimensionality reduction, compression of data and parameters, prevention of overfitting, and improvement of the model’s robustness. We used max pooling layers when constructing the IGRNet model, placing them after convolution layers 2 and 4; then, we used a 2 × 2 sampling window to control the shrinking speed of the feature graph. The fully connected layer purifies the features learned by the convolutional layers and maps the learned “distributed feature representation” to the sample marker space. In this study, a fully connected layer was added after the final pooling layer. The softmax function was introduced into the output layer of the model, and the output value was converted to a relative probability. To summarize, the overall architecture of our IGRNet proceeds as follows: input layer -- convolution layer -- convolution layer -- pooling layer -- convolution layer -- convolution layer -- pooling layer -- fully connected layer -- output layer.

#### 2.3.2. Nonlinear Activation Function in IGRNet

Nonlinear activation functions can change the linear combination of the network, allowing it to approximate any nonlinear function arbitrarily. To determine the optimal activation function in the present study, we compared four activation functions widely used in deep learning fields: ReLU, LeakyReLU, ELU, and Clipped ReLU. Here, the ReLU function is expressed as

(1) f(x)=max(0,x)

This function maps the input into two segments. If the input value is less than zero, the original value is mapped to zero; if the input value is greater than zero, the original value is retained. That is, when calculating forward, a large number of features will be lost. As can be seen from the derivative, the gradient is preserved when calculating backwards. Thus, when the input is a positive number, no gradient saturation problems occur. However, when the input is negative, ReLU is completely inactive; thus, once a negative number is entered, ReLU will cease to function. In contrast, LeakyReLU assigns a non-zero slope to all negative values. The corresponding mathematical expression is:

(2) $$f(x)=\left\{ \begin{array}{l} x,x>0\\ leak*x,x\leq 0 \end{array}\right.$$

The unique feature of this function is that the negative axis maintains a small constant *leak*, such that when the input information is less than zero, the information is not completely lost but is retained accordingly. That is, ReLU features no gradient in the regions below zero, whereas LeakyReLU features a very small gradient in this region. In this experiment, we repeatedly evaluated the *leak* and found that when it as set to 0.0001, that the model achieved optimal performance. The ELU function was also used in this study. Its expression is:

(3) $$f(x)=\left\{ \begin{array}{l} x,x\geq 0\\ \alpha (e^{x}-1),x<0 \end{array}\right.$$

where α is a constant. It can be seen that the left-hand side (the regions below zero) of the function exhibits soft saturation, whereas the right-hand side (the regions above zero) has no saturation. The linear term on the right-hand side means that the ELU mitigates the disappearance of the gradient, and the soft saturation feature on the left-hand side makes it more robust to input changes or noise. ClippedReLU is an improvement of ReLU, in which an upper limit *ceiling* has been added. The corresponding function formula is:

(4) $$f(x)=\left\{ \begin{array}{l} 0,x<0\\ x,0\leq x<ceiling\\ ceiling,x\geq ceiling \end{array}\right.$$

Here, the activation range of ReLU is limited to encourage the model to learn sparse features earlier. If left unrestricted, the increase in activation value may cause precision loss. Our repeated tests showed that IGRNet performed best when *ceiling* was set as 6.

#### 2.3.3. Batch Normalization (BN) in IGRNet

To improve the neural network efficacy, we introduced a BN layer after each convolutional layer. The BN algorithm [30] has been shown to accelerate the training speed of models and improve their generalizability, owing to its powerful functions. Using that, our model can recover the feature distribution to be learned by the original network, this is useful for parameter adjustments.

#### 2.3.4. Dropout in IGRNet

**Figure 3:**
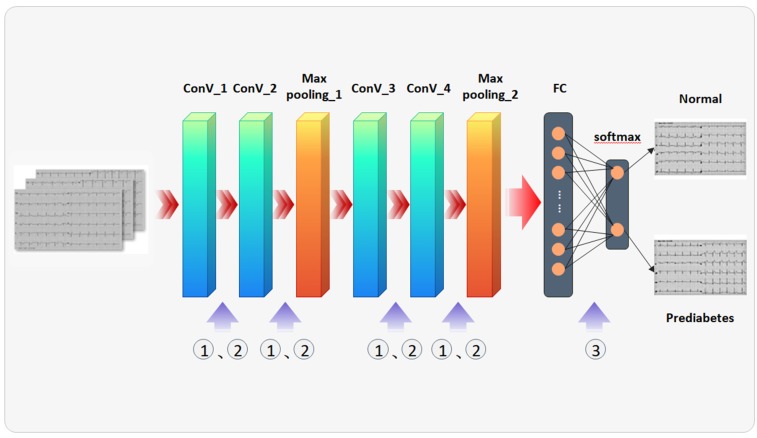
IGRNet architecture settings: ① represents batch normalization; ② represents activation functions, including ReLU, LeakyReLU, ELU, and Clipped ReLU; and ③ represents the dropout function.

Traditional CNNs have weak generalizability and are prone to overfitting when insufficient data are provided. In view of the small number of ECGs in this experiment, a dropout layer was introduced after the fully connected layers to prevent overfitting. In our experiment, the best value was 0.5. Therefore, the model architecture of IGRNet is as shown in Figure 3.

### 2.4. Mainstream Convolutional Neural Networks

To assess the performance of the IGRNet mentioned above, we introduced two mainstream CNNs (AlexNet and GoogLeNet) commonly used in the field of ECG recognition. It must be emphasized that both CNNs had been pre-trained with 1000 classification databases in ImageNet, and this study performed transfer learning on both.

AlexNet [31] has been widely used in the field of image recognition, having been successfully employed many times in the diagnostic study of ECGs [32,33]. In this study, the number of neurons in the output layer was changed to two, and the final layer was updated based on the initialization model.

GoogLeNet has been exploited in the field of medical image recognition [34,35] due to its excellent performance. Our experiments used an improved Inception module unit, which was stacked with nine Inception modules (we adopted the GoogLeNet V1 structure); furthermore, we replaced the last three layers of the network and added the new layers “Fully connected layer”, “softmax layer”, and “classification output layer” to the hierarchy, based on the pre-trained model. Meanwhile, in order to ensure that the new layer learned faster than the transport layer, we increased the learning factor of the fully connected layer and then connected the last transport layer (pool5-drop_7x7_s1) in the network to the new layer, to complete the architecture of the transfer model.

### 2.5. Baseline Algorithms

The HOG feature was first adopted for static pedestrian detection [36]; then, Rathikarani et al. [37] used it to extract image features from ECGs, and classified three heart diseases: arrhythmia, myocardial infarction, and conduction block. SVM, RF, and K-NN, as classical machine learning algorithms, were also used in early studies of ECGs [38,39,40]. In the baseline method of this study, we employed the HOG algorithm to extract the image features from the 12-lead ECG and trained the aforementioned three classifiers for IGR diagnosis. We modified the image size to 200 × 200 and set the cell to 32 × 32 pixels, with each block containing 2 × 2 cell units. Thus, 900-dimension feature vectors were extracted from each ECG, and all the vectors were input into the classifier for training.

## 3. Experiment

### 3.1. Experimental Setup

The operating system used in this study was Windows 10, and the computer was configured with an Intel Core I7-6700HQ 3.5 GHz central processing unit, and 4 GB of memory. This experiment adopted MATLAB R2018a and Python 3.8 to complete the tasks in this paper. In this study, a 5-fold cross-validation was performed on all six training sets; that is, each dataset was evenly divided into five sub-datasets, from which we randomly selected four as the training set, and the remaining one as the validation set. In addition, the IGRNet, AlexNet, GoogLeNet, and baseline algorithms were optimized during the training process. An Adam optimization algorithm was used. After cross-validation, the performing models were adopted to test on the independent test sets to verify their capabilities.

### 3.2. Experimental Process

Our experimental work included the following five sections:

1. **Experiment #1** For dataset_1, experiments were conducted on the four aforementioned activation functions for IGRNet, so as to find the activation function with the optimal performance and thus improve the generalizability of the model. The four activation functions were optimized in the preliminary experiments. Additionally, the InitialLearnRate was set to 0.0001, the L2Regularization was set to 0.001 during training.
2. **Experiment #2** To verify the superiority of the IGRNet architecture in the task of ECG prediabetes diagnoses, we compared it with two mainstream CNN models (AlexNet and GoogLeNet) on dataset_1. All models were optimized during training.
3. **Experiment #3** The adjusted SVM, RF, and K-NN models were also compared against IGRNet, to verify the superiority of the 2D-CNN proposed in this paper.
4. **Experiment #4** To reduce the interference of other factors on the ECG diagnosis and further improve the performance of the model, IGRNet was used to perform cross-validation on dataset_2, dataset_3, dataset_4, dataset_5, and dataset_6.
5. **Experiment #5** In order to verify the true performance of IGRNet in IGR diagnosis, we employed the model trained by the former (dataset_1-6) to test independent test set_0-6 respectively. In addition, in order to more strictly prove the superiority of the model proposed in this paper, we also tested other models and different activation functions on the total independent test set.

### 3.3. Experimental Evaluation

In our experiment, the average values and the corresponding 95% confidence intervals (CIs) of 5-fold cross-validation were adopted to pick out the best models from the methods mentioned above. We considered accuracy (Acc), sensitivity (Sens), specificity (Spec), and precision (Prec) as the evaluation criteria. The corresponding calculation formulas are as follows. Meanwhile, the area under the receiver operating characteristic curve (AUC) and training or testing time was also considered.

(5) $$\mathrm{Acc}=\frac{TN+TP}{TN+TP+FN+FP}$$

(6) $$\mathrm{Sens}=\frac{TP}{TP+FN}$$

(7) $$\mathrm{Spec}=\frac{TN}{TN+FP}$$

(8) $$\mathrm{Prec}=\frac{TP}{TP+FP}$$

TP (positive) refers to the proportion of correct classifications for positive samples, FP (false positive) refers to the proportion of incorrect classifications indicating that a sample belongs to a specific category, TN (negative) refers to the proportion of correct classifications of negative samples, and FN (false negative) refers to the proportion of incorrect classifications indicating that a sample does not belong to a specific category.

## 4. Results and Discussion

### 4.1. Selection of Activation Functions

**Figure 4:**
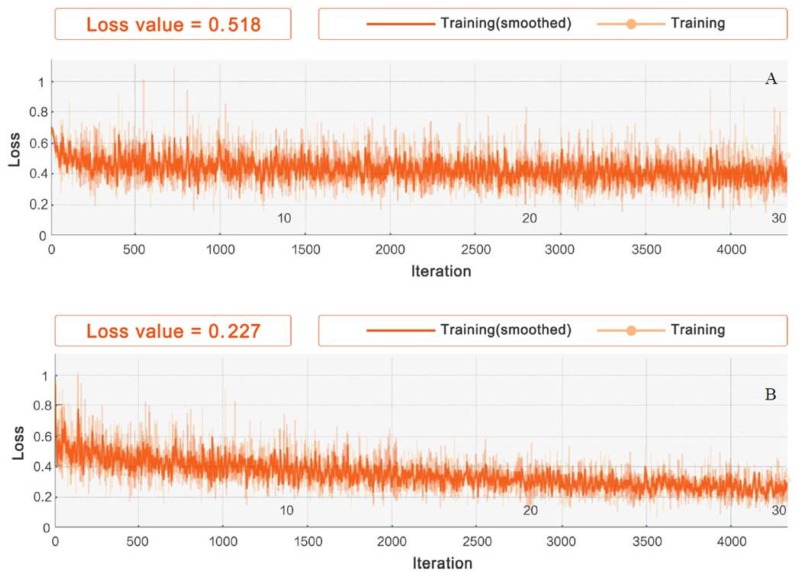

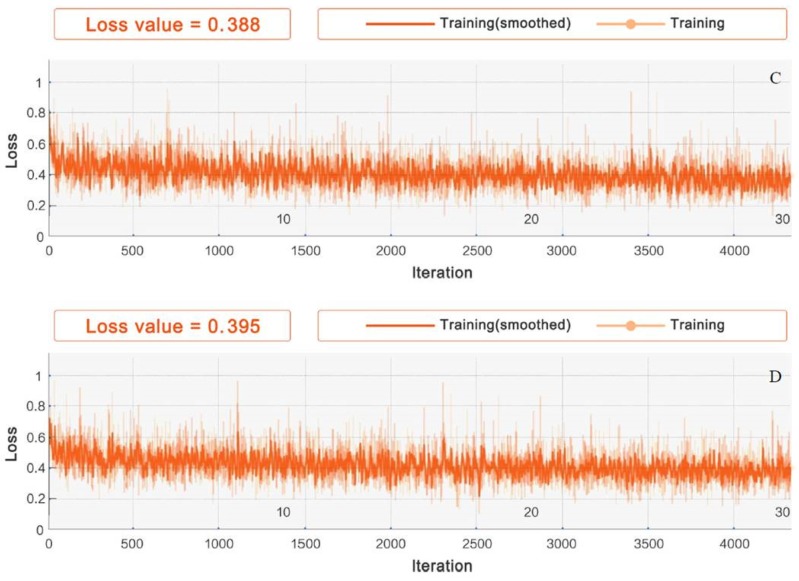
Iterative processes of different activation functions in IGRNet: (**A**) is the loss change of the ReLU function, (**B**) is the loss change of the LeakyReLU function, (**C**) is the loss change of the ELU function, and (**D**) is the loss change of the ClippedReLU function.

The choice of nonlinear activation function is crucial to the performance of the model. At present, there is no definitive conclusion on the performance of the activation functions in the field of deep learning. The applicability of different activation functions to different datasets requires further investigation. In this study, we performed experiments using ReLU, LeakyReLU, ELU, and Clipped ReLU on dataset_1, with *leaky* set to 0.0001 and *ceiling* set to 6. The model stabilized after 4380 iterations of IGRNet. At this point, the training was stopped and verified. The change in model loss during training is shown in Figure 4.

It can be seen that LeakyReLU’s loss change is the most notable and that the final loss value is smallest (0.227), thus this function achieves excellent performance in this study. We also evaluated the models using various activation functions for 5-fold cross-validation, as shown in Table 4.

**Figure 5:**
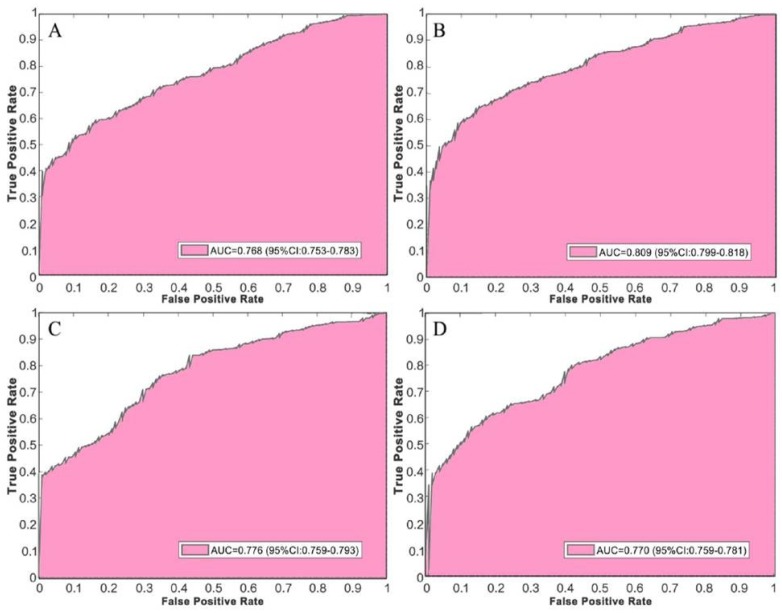
ROC curves of IGRNet using various activation functions: (**A**) is the ROC curve of the model using the ReLU function, (**B**) is the ROC curve of the model using the LeakyReLU function, (**C**) is the ROC curve of the model using the ELU function, and (**D**) is the ROC curve of the model using the ClippedReLU function.

The average accuracy of IGRNet using LeakyReLU is 0.854, its sensitivity is 0.862, its specificity is 0.865, and its precision is 0.895; thus, it is seen to relatively outperform the other three activation functions. Figure 5 shows the receiver operating characteristic (ROC) curves and corresponding AUC values of the model using each activation function. The average AUC of the model using LeakyReLU is 0.809, which is still higher than is seen using ReLU (0.768), ELU (0.776), and ClippedReLU (0.770).

### 4.2. Comparison with Deep Convolutional Neural Networks

**Figure 6:**
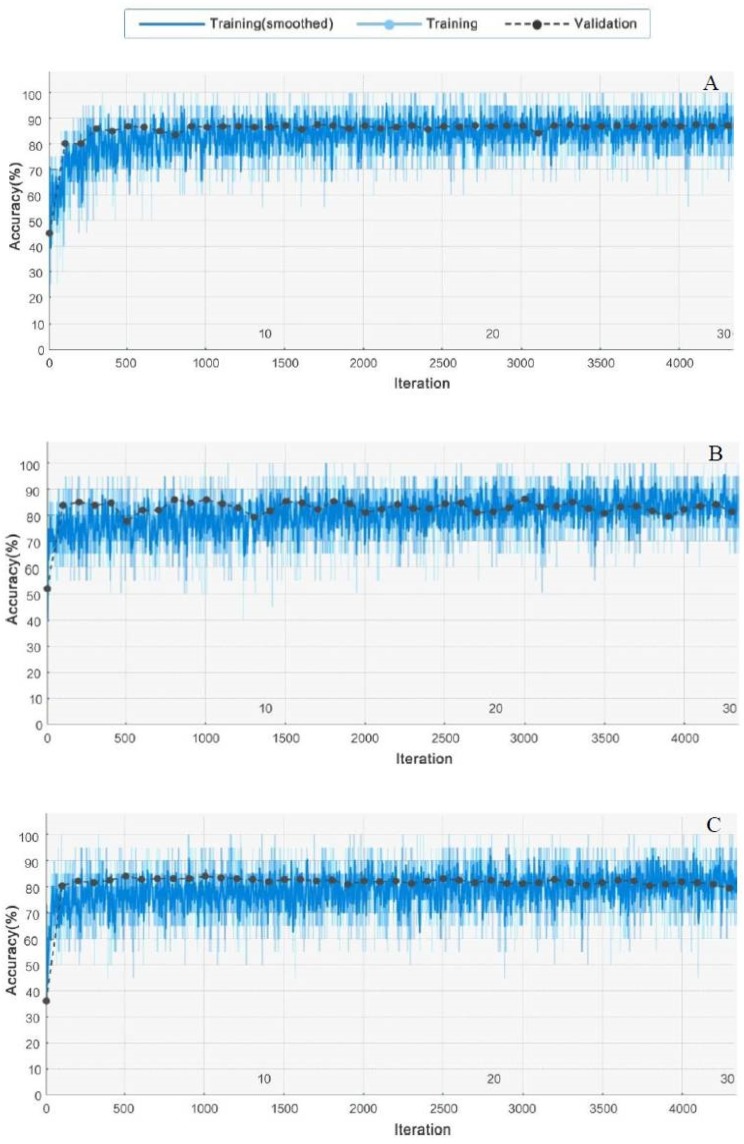
Training and verification processes for IGRNet, AlexNet, and GoogLeNet: (**A**) is the training result of IGRNet, (**B**) is the training result of AlexNet, and (**C**) is the training result of GoogLeNet.

In addition to IGRNet, our experiment also considered the 5-fold cross-validation of AlexNet and GoogLeNet under the same number of iterations. The training processes of the three CNN models are shown in Figure 6. Although AlexNet and GoogLeNet have complex architectures and deeper network layers, their performance on dataset_1 is inferior to that of IGRNet.

**Figure 7:**
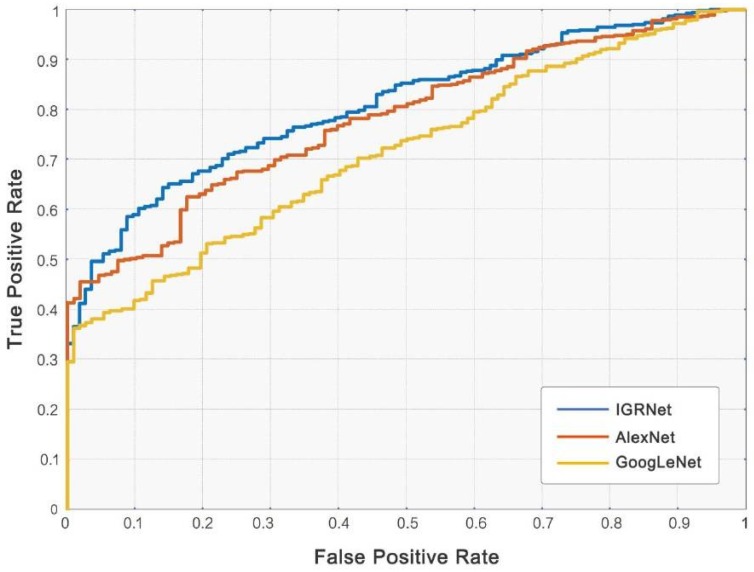
ROC curves of convolutional neural networks (CNNs).

We calculated the evaluation indicators of mainstream CNN models after training and compared them with IGRNet (see Table 5). Each training time of CNNs was also calculated to evaluate their work efficiency. The average diagnostic accuracies of AlexNet and GoogLeNet (0.807 and 0.820) are inferior to that of our proposed model. The AUC values of the two mainstream models and IGRNet also differ (the corresponding ROC curves as shown in Figure 7). On the other hand, IGRNet’s training cost is less than mainstream CNNs due to its architecture.

### 4.3. Comparison with Baseline Algorithms

**Figure 8:**
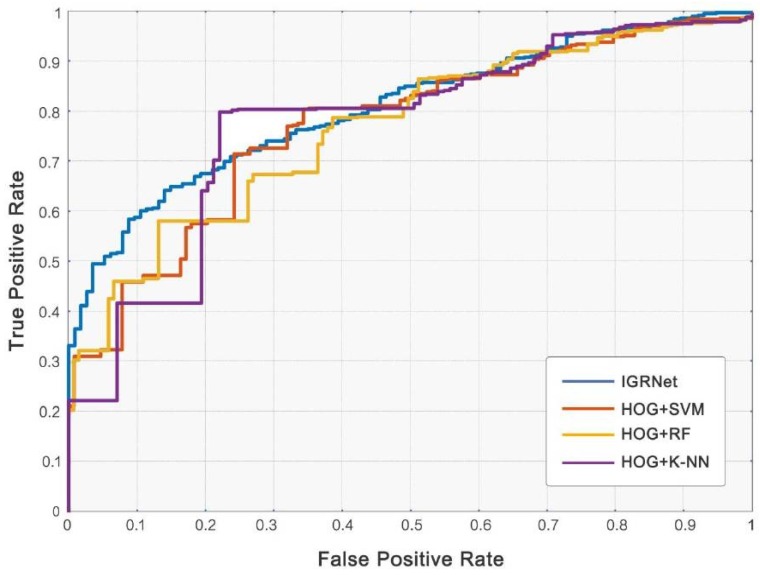
ROC curves of IGRNet and baseline algorithms.

After the HOG feature extraction, the baseline algorithms of SVM, RF, and K-NN were used to classify ECG images and thus evaluate our model. From the perspective of accuracy, sensitivity, specificity, precision, and AUC (corresponding results are shown in Table 6 and Figure 8), K-NN performs best of the baseline algorithms, achieving average value of 0.824, 0.718, 0.904, 0.891, and 0.775, respectively. However, it still lags behind IGRNet. From the perspective of training time, traditional machine learning algorithms take less time than IGRNet, so training costs are lower.

### 4.4. Further Improvement

According to previous studies, the ECGs of patients are affected by personal factors, including weight, gender, and age, which may influence the model efficacy in the diagnosis of prediabetes. Therefore, we employed IGRNet to conduct further experiments on training sets that had been grouped according to weight, gender, and age. The results are shown in Table 7. From the verification results for different datasets, it can be seen that the performances of categorized models are improved to a certain extent compared to that of the unclassified model. Among these categorized models, the IGR diagnosis of dataset_2 and dataset_3 for the same weight range is clearest. When the datasets contain only normal-weight people (BMI < 25.0), the average validation accuracy is 7.30% higher than the initial level, and the AUC is 5.20% higher. When all the datasets contain overweight subjects (BMI ≥ 25.0), the average validation accuracy increases by 6.00% and the AUC increases by 4.50%.

### 4.5. Test Performance on Independent Test Sets

It is undeniable that cross-validation after data augmentation may divide the augmented images from the same raw image into the training set and the validation set at the same time, making the model catch the specific pattern in similar images. This may cause artificially inflate model performance. Therefore, we must test them on independent test sets to reflect the true strength of IGRNet. In the experiment, IGRNet trained with dataset_1–6 were employed to test independent test set_0-6 (the model trained on dataset_1 was adopted to test test set_0–1), respectively, and the results are shown in Table 8. We found that IGRNet’s test performance has a certain decrease compared to the cross-validation results. However, undoubtedly, the performance on the classified test set is still better than the mixed test set that is similar to the total test set. Among them, when the test set contains only normal-weight people (BMI < 25.0), the diagnostic accuracy is 0.856 and the AUC is 0.825. When the test set contains only overweight subjects (BMI ≥ 25.0), the diagnostic accuracy is 0.850 and the AUC is 0.808. Moreover, IGRNet’s recognition time of each ECG image is about 0.160–0.190 s with the help of existing equipment, which is expected to realize its advantages of real-time diagnosis.

To rigorously prove that IGRNet using leakyReLU possesses the best performance compared to other activation functions, the experiment employed the model with various activation functions (ReLU, LeakyReLU, ELU, and ClippedReLU) to test the total independent test set and the results are shown in Table 9. It is not difficult to find that the conclusion obtained from these results is consistent with the previous validation experiment. Additionally, the aforementioned mainstream CNNs and traditional machine learning models are also employed on the total independent test set to compare the performance of IGRNet in the condition of real data. The corresponding results are shown in Table 10. Consistent with the cross-validation conclusion, the results on the independent test set show the excellent performance of IGRNet.

### 4.6. Discussion

In this paper, for the first time, it was found that 2D-CNN can be used to diagnose IGR non-invasively and in real-time, by using 5-s 12-lead ECGs. After training, validation, comparison, and testing, our proposed IGRNet was seen to effectively identify the corresponding ECGs of prediabetic patients, providing a new method for clinical diagnosis of this disease in the future.

In this study, we conducted 5-fold cross-validation on ECG images after performing data augmentation operations. It is worth emphasizing that the purpose of the validation experiment in this paper is mainly three, one is to compare the relative performance of IGRNet using various activation functions to select the superior activation function, the second is to compare the relative performance among mainstream CNNs, conventional machine learning algorithms, and IGRNet, and the third is to provide experimental support for improved models. Considering that the validation set does not have sufficient generalized representativeness due to the generation of derived data in the above process, we introduced 663 independent samples as the test sets to strictly test the real performance of each model. The results show that the conclusions obtained from independent testing are consistent with cross-validation, which indicates that the validation experiment provides evidence for model selection and the test experiment evaluates the true strength of the models.

The nonlinear activation function has a large impact on the performances of the deep learning models. Appropriate activation functions can effectively improve the performance of the model. However, no conclusion has yet been made on the performance of mainstream activation functions, and further experimental research is required. Zhong et al. [41] used CNN to detect fetal QRS complexes through non-invasive fetal electrocardiograms, they found that the ReLU function performed best in this task after comparing multiple activation functions. Zha et al. [33] found that Swish and ELU functions performed better in ECG classification using one one-dimensional CNN (1D-CNN). In our experiment, after comparing ReLU, LeakyReLU (*leak* = 0.0001), ELU, and ClippedReLU (*ceiling* = 6) in IGRNet, LeakyReLU was found to be optimal, this may be related to the small slope of the output under a negative input. Because the derivative was never zero, the occurrence of silent neurons was reduced, and gradient-based learning was facilitated. Therefore, the problem of neurons being unable to learn after the ReLU function enters a negative interval was solved.

Furthermore, by comparing the deep transfer learning models of AlexNet and GoogLeNet, as well as the SVM, RF, and K-NN algorithms after HOG feature engineering, we found that IGRNet using only four convolutional layers obtained optimal recognition for the task in this study. In terms of the key analysis of image features extracted by CNN, we suspect that the texture features of the 12-lead ECG were relatively uniform, and the features extracted by the deep mainstream CNNs were too deep, resulting in over-fitting. The architecture of IGRNet was more suitable for this task. In terms of training cost, the training time of IGRNet is shorter than mainstream CNNs, which is convenient for further development in the future. On the other hand, traditional machine learning has the disadvantage of manual feature extraction, which cannot fully reflect the details of ECG features, resulting in a diagnosis efficacy inferior to that of IGRNet.

Considering weight, gender, and age to be potential factors affecting the ECG, we classified the data, and the model evaluation values of the final improved method on the validation sets and independent test sets were relatively improved compared to those of the original model, indicating that weight, gender, and age affect the judgment of IGR patients by AI. On the other hand, our results were consistent with previous conclusions that the above factors influence the ECG changes to a certain extent. It is worth noting that Alpert et al. [25] found that overweight or obese subjects can exhibit a variety of ECG changes, including a left shift of the p-wave, QRS, and t-wave axes. In our study, IGRNet showed the most significant improvement for people with the same body weight range (BMI ≥ 25.0 or BMI < 25.0), which suggests that body weight has a larger influence on ECG than the other two. Thus, the model of controlling for weight factors can be employed to diagnose prediabetes with improved accuracy.

It must be emphasized that the current study highlights a certain difference between prediabetic and normal people in ECGs; however, this difference is usually ignored in clinical practice. Among them, Balcıoğlu et al. found that IGR patients exhibited different degrees of CAND, by recording the heart rate variability and heart rate turbulence indexes of 24-h dynamic ECGs [42]. Yu et al. adopted dual-source computed tomography to evaluate the relationship between coronary atherosclerosis (CAA) and blood glucose levels; they found that the prevalence of CAA in the prediabetic group was slightly higher than that of the normoglycemic group but lower than that of the diabetic group, which indicates that prediabetic patients have an increased risk of CAA. However, it is difficult to distinguish clinical symptoms from those of normal blood glucose [43]. We developed a deep learning model, IGRNet, based on the results of previous studies. The results of the independent test sets show that the highest detection accuracy of this model reaches 0.856, and the average recognition time of an ECG image is only 0.160–0.190 s. Therefore, this AI model is expected to carry out highly accurate, convenient, non-invasive, and real-time diagnoses of prediabetes by identifying the ECG characteristics of IGR patients. Our research differed from previous studies, which used ECG signals to directly identify different types of heart disease; instead, it represents a new attempt to diagnose IGR by extracting ECG changes that are characteristic of prediabetes. 

In recent years, a number of studies have been conducted on non-invasive blood glucose monitoring, for which the ideal technology to use is near-infrared spectroscopy for non-invasive blood glucose detection [44,45]. Even so, there is still no non-invasive blood glucose detection method that meets the clinical detection accuracy. In terms of spectral analysis: First, effective spectral signals are weak owing to the low blood sugar content of the human body; furthermore, they are susceptible to interference from other signals and have a low signal-to-noise ratio. Second, temperature, humidity, and other conditions of the measurement site directly affect the transmission of light and reduce the detection accuracy. Moreover, the environment of the human body is complex and diverse, and the absorption of other physiological components overlaps with that of glucose; thus, the influence of its concentration on the light intensity is even larger than the influence of changes in the glucose concentration. Instead, the ECG, which reflects physiological changes in the body in real-time, is unaffected by external factors, providing a novel detection method. This research can be used for portable ECG-monitoring equipment, creating the possibility for highly accurate clinical detection in the future.

However, it should be noted that our research also contains some defects. First of all, cross-validation after data augmentation on the total training set may lead to artificial accuracy improvement, because part of the derived data may be divided into validation sets, which need to enhance the reliability of validation results in the future work. Next, this study adopted retrospective data, which has an inherent flaw, that is, the assurance may not reach the ideal situation. The ECG is not affected by external interference, but is affected by subjects’ personal factors. In this paper, due to limited sample information, we only considered weight, gender, and age; however, there are more factors affecting the ECG. Meanwhile, the above factors cannot be jointly constrained due to insufficient sample size. Furthermore, our method is limited by the population, which meets the condition of having “no history of cardiovascular diseases.” Additionally, the 5-s 12-lead ECG may not truly reflect the ECG status of the human body, which may lead to misdiagnosis and missed diagnoses. Moreover, our study only focused on the clinical diagnosis of IGR, and thus could not monitor blood glucose concentration. In future, the datasets should be expanded to classify a range of blood glucose types in the human body at a wider range of levels. Additionally, the characteristics of “black box” to deep learning make IGRNet’s work not transparent enough.

## 5. Conclusions

In view of the characteristics of prediabetes, which presents no obvious clinical symptoms and is easy to neglect, this paper proposed the use of deep learning for diagnoses from human ECGs; this requires only a 5-second 12-lead ECG and is characterized as a highly accurate, non-invasive, and real-time procedure. By comparing with mainstream CNNs and traditional machine learning techniques, it was found that the IGRNet model designed in our study obtained an optimal diagnostic performance, with a maximum accuracy of 0.856, a sensitivity of 0.839, a specificity of 0.902, a precision of 0.887, and an AUC of 0.825. To our knowledge, this is the first study to report that AI can efficiently identify prediabetes from ECGs. It has the potential to be clinically promoted in the future due to its outstanding performance in this task.

## Figures and Tables

**Table 1:** The composition of the training sets in this paper.

Dataset Name	Prerequisite	Number of Samples (Normal)	Number of Samples (IGR)
dataset_1	Total	1750	501
dataset_2	BMI ≥ 25	643	282
dataset_3	BMI < 25	1107	219
dataset_4	Men	1043	361
dataset_5	Women	707	140
dataset_6	Age < 60	1673	433
dataset_7	Age ≥ 60	77	68

Notes: Because the dataset_7 data volume is too small to support subsequent experiments, this study used the remaining datasets.

**Table 2:** The composition of the independent test sets.

Dataset Name	Prerequisite	Number of Samples (Normal)	Number of Samples (IGR)
test set_0	Total	503	160
test set_1	Mixed	250	100
test set_2	BMI ≥ 25	228	73
test set_3	BMI < 25	275	87
test set_4	Men	269	89
test set_5	Women	234	71
test set_6	Age < 60	442	101
test set_7	Age ≥ 60	61	59

Notes: 1. In order to ensure that the total number of positive and negative samples in comparative experiments was approximately the same, some ECG samples were randomly selected from the overall independent test set to form test set_1. 2. Because this research did not consider the experiment on dataset_7, the test set_7 was not adopted.

**Table 3:** The composition of the training sets after data augmentation in this paper.

Dataset Name	Prerequisite	Number of Samples (Normal)	Number of Samples (IGR)
dataset_1	Total	1750	1503
dataset_2	BMI ≥ 25	1929	1692
dataset_3	BMI < 25	1660	1533
dataset_4	Men	1564	1444
dataset_5	Women	1414	1400
dataset_6	Age < 60	1673	1299

**Table 4:** Evaluation of IGRNet using various activation functions. The values represent the average values of the verification results and their 95% CIs.

Activation Function	Acc	Sens	Spec	Prec
ReLU	0.795 (0.785–0.805)	0.763 (0.759–0.768)	0.849 (0.843–0.854)	0.887 (0.877–0.896)
LeakyReLU	0.854 (0.839–0.870)	0.862 (0.853–0.871)	0.865 (0.857–0.874)	0.895 (0.882–0.907)
ELU	0.839 (0.830–0.847)	0.842 (0.839–0.846)	0.854 (0.846–0.862)	0.882 (0.874–0.890)
ClippedReLU	0.819 (0.803–0.835)	0.795 (0.770–0.820)	0.898 (0.887–0.909)	0.925 (0.911–0.939)

**Table 5:** The results of comparison with deep CNNs. The values represent the average values of the results and their 95% CIs.

CNN Model	Acc	Sens	Spec	Prec	AUC	Training Time (s)
IGRNet	0.854 (0.839–0.870)	0.862 (0.853–0.871)	0.865 (0.857–0.874)	0.895 (0.882–0.907)	0.809 (0.799–0.818)	940.6 (901.1–980.1)
AlexNet	0.807 (0.792–0.822)	0.780 (0.753–0.807)	0.904 (0.886–0.922)	0.921 (0.890–0.952)	0.787 (0.777–0.797)	6477.2 (6341.8–6612.6)
GoogLeNet	0.820 (0.802–0.838)	0.752 (0.719–0.786)	0.924 (0.907–0.941)	0.906 (0.891–0.921)	0.716 (0.698–0.733)	8948.5 (8761.4–9135.6)

**Table 6:** The results of comparison with baseline algorithms. The values represent the average values of the results and their 95% CIs.

Classification Method	Acc	Sens	Spec	Prec	AUC	Training Time (s)
IGRNet	0.854 (0.839–0.870)	0.862 (0.853–0.871)	0.865 (0.857–0.874)	0.895 (0.882–0.907)	0.809 (0.799–0.818)	940.6 (901.1–980.1)
HOG+SVM	0.809 (0.795–0.822)	0.720 (0.703–0.737)	0.867 (0.836–0.899)	0.836 (0.803–0.868)	0.772 (0.764–0.780)	95.7 (87.5–103.9)
HOG+RF	0.800 (0.774–0.827)	0.687 (0.670–0.704)	0.836 (0.794–0.878)	0.842 (0.826–0.859)	0.764 (0.749–0.780)	98.3 (93.8–102.8)
HOG+K-NN	0.824 (0.805–0.844)	0.718 (0.698–0.739)	0.904 (0.878–0.929)	0.891 (0.867–0.915)	0.775 (0.768–0.782)	84.8 (77.1–92.5)

**Table 7:** Experimental results on different datasets. The values represent the average values of the results and their 95% CIs.

Dataset	Acc	Sens	Spec	Prec	AUC
dataset_2	0.914 (0.891–0.937)	0.918 (0.899–0.937)	0.895 (0.875–0.915)	0.911 (0.895–0.927)	0.854 (0.845–0.863)
dataset_3	0.927 (0.916–0.938)	0.882 (0.853–0.911)	0.967 (0.962–0.972)	0.960 (0.949–0.971)	0.861 (0.838–0.884)
dataset_4	0.869 (0.848–0.890)	0.785 (0.780–0.790)	0.916 (0.904–0.928)	0.920 (0.902–0.938)	0.844 (0.829–0.859)
dataset_5	0.878 (0.865–0.891)	0.814 (0.800–0.828)	0.961 (0.955–0.967)	0.956 (0.934–0.978)	0.851 (0.831–0.871)
dataset_6	0.888 (0.869–0.907)	0.755 (0.752–0.758)	0.980 (0.974–0.986)	0.959 (0.950–0.968)	0.858 (0.834–0.882)

**Table 8:** Experimental results using IGRNet on the independent test sets.

Dataset	Acc	Sens	Spec	Prec	AUC	Test Time (s)
test set_0	0.778	0.808	0.775	0.852	0.773	101.2
test set_1	0.781	0.798	0.789	0.846	0.777	57.7
test set_2	0.850	0.834	0.820	0.879	0.808	56.4
test set_3	0.856	0.839	0.902	0.887	0.825	58.3
test set_4	0.821	0.760	0.925	0.901	0.801	58.4
test set_5	0.833	0.800	0.907	0.888	0.794	57.2
test set_6	0.829	0.697	0.892	0.874	0.788	85.9

**Table 9:** Experimental results using IGRNet with various activation functions on the total independent test set.

Activation Function	Acc	Sens	Spec	Prec	AUC
ReLU	0.739	0.687	0.765	0.819	0.742
LeakyReLU	0.778	0.808	0.775	0.852	0.773
ELU	0.765	0.784	0.809	0.822	0.764
ClippedReLU	0.756	0.799	0.780	0.834	0.761

**Table 10:** Experimental results using different machine learning models on the total independent test set.

Model	Acc	Sens	Spec	Prec	AUC	Test Time (s)
IGRNet	0.778	0.808	0.775	0.852	0.773	101.2
AlexNet	0.749	0.770	0.821	0.862	0.755	117.6
GoogLeNet	0.754	0.693	0.837	0.846	0.689	125.1
HOG+SVM	0.736	0.698	0.768	0.840	0.757	13.5
HOG+RF	0.741	0.685	0.755	0.853	0.752	18.8
HOG+K-NN	0.760	0.705	0.799	0.837	0.761	11.7
